# Oral Anticoagulation Discontinuation Following Catheter Ablation of Typical Atrial Flutter

**DOI:** 10.19102/icrm.2021.120703

**Published:** 2021-07-15

**Authors:** Bilal M. Alqam, Kirby N. Von Edwins, Subodh Devabhaktuni, Hakan Paydak, Naga Venkata K. Pothineni

**Affiliations:** ^1^Department of Cardiovascular Medicine, University of Arkansas for Medical Sciences, Little Rock, AR, USA

**Keywords:** Atrial flutter, bleeding, catheter ablation, oral anticoagulation, stroke

## Abstract

Catheter ablation (CA) of typical atrial flutter (AFL) is the preferred treatment for typical AFL due to its excellent long-term success rate. However, current guidelines recommend pursuing oral anticoagulation (OAC) based on established indices of stroke regardless of the perceived success of ablation. We conducted a retrospective study of all patients who underwent typical AFL ablation at our institute from 2011 to 2017. All patients continued OAC for at least six weeks post-CA and underwent 24-hour Holter monitoring. OAC was discontinued if there was no evidence of recurrence at six weeks. In patients with low left ventricular ejection fraction or prior atrial fibrillation episodes, OAC was continued for six months with repeat Holter monitoring at six months. A total of 106 patients were included in our analysis, with a mean age of 64 ± 14 years and 78.3% of whom were male. The mean CHA_2_DS_2_-VASc score was 3 ± 1 points. OAC was discontinued by six weeks in 17% and at one year in 55.7% of patients, respectively, but was continued indefinitely in 44.3%. Over a mean follow-up period of 28.6 ± 27.3 months, there was one ischemic stroke in the OAC discontinuation group and no ischemic events in the continued OAC group. There were a total of three major bleeding events, all in the OAC group. In patients undergoing successful AFL ablation, a strategy of OAC discontinuation with close rhythm monitoring appears feasible. The benefit of continued OAC in this cohort may be outweighed by an adverse risk of bleeding. Further studies examining rhythm-guided OAC can minimize unnecessary exposure to long-term anticoagulation.

## Introduction

Cavotricuspid isthmus-dependent atrial flutter (AFL) is a commonly encountered arrhythmia and a well-recognized risk factor for cardioembolic stroke. Catheter ablation (CA) is the preferred treatment approach for typical AFL due to its excellent long-term success rate. Oral anticoagulation (OAC) therapy is a mainstay to reduce stroke risk in patients with AFL, but optimal OAC strategies following successful CA remain unclear. Current guidelines recommend OAC based on established indices of stroke risk, such as the CHA_2_DS_2_-VASc score, regardless of the perceived success of ablation.^1^ In addition, these recommendations are based mainly on studies on atrial fibrillation (AF) rather than AFL. A strategy of anticoagulation discontinuation after successful AFL ablation has not been well studied, even though similar strategies have been shown to be safe and effective following successful AF ablation.^2^ We sought to examine the outcomes of a strategy of OAC discontinuation following successful AFL ablation.

## Methods

We conducted a retrospective study of all patients who underwent CA of typical AFL at our institution from 2011 to 2017 using our electrophysiology laboratory database. Patients who underwent successful ablation for typical AFL were included. After obtaining approval from the institutional review board, each chart was manually reviewed by one of our investigators and the following information was collected: demographic data, comorbidities, medications used, type of AFL, complications within the first 30 days, Holter monitor results at one and six months, time to recurrence of AFL or AF, and outcomes.

Our institutional protocol involved OAC for at least six weeks after successful AFL ablation, after which patients underwent 24-hour Holter recording. In the absence of AF or AFL, OAC was discontinued. In patients with a prior history of AF or heart failure [left ventricular (LV) ejection fraction < 50%], OAC was continued for six months postablation and a Holter recording was obtained. If Holter monitoring at six months showed no evidence of AF/AFL, OAC was discontinued. During long-term follow-up, Holter monitoring was performed annually. OAC was restarted in patients who experienced a recurrence of AFL /AF during follow-up. The primary study outcome was the rate of OAC discontinuation and the occurrence of major adverse events (eg, thromboembolic events, major bleeding).

Patients were classified into two groups based on whether OAC was stopped or continued after the ablation procedure. Categorical variables are reported as counts and percentages; differences were assessed using the chi-squared test. Continuous variables are presented as mean with one standard deviation values; differences were compared using a two-sample Student’s t-test. All statistical analyses were performed using the Statistical Package for the Social Sciences version 25.0 software program (IBM Corp., Armonk, NY, USA).

## Results

**Figure 1:**
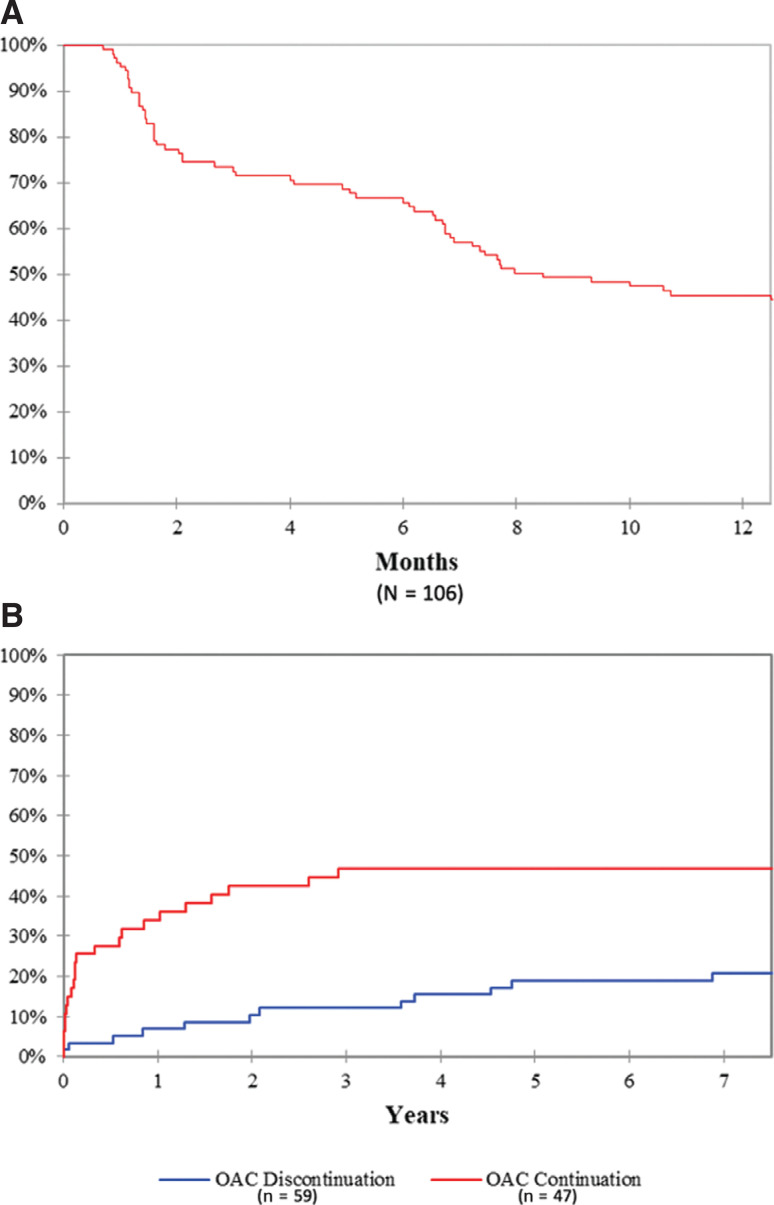
**A:** Percentage of patients remaining on OAC following successful AFL ablation. **B:** Cumulative incidence of new-onset AF after AFL ablation. AF: atrial fibrillation; OAC: oral anticoagulation.

A total of 106 patients underwent AFL ablation during the study period and were included in this analysis (mean age: 64 ± 14 years; 78.3% male). The mean CHA_2_DS_2_-VASc score was 3 ± 1 points. A total of 67.9% of patients had no prior history of AF; of these, 3.8% had AF detected during six-week Holter monitoring. OAC (42.5% on warfarin) was continued for at least four weeks post-CA in all patients. OAC was discontinued by six weeks in 17% of patients, by six months in 32.1% of patients, and by one year in 55.7% of patients **(Figure 1A)** and was continued indefinitely in 44.3% of patients. The most common reasons for continued OAC included a history of AF, new-onset AF during follow-up, or other nonarrhythmic indications for OAC. Over a mean follow-up period of 28.6 ± 27.3 months, 2.8% of patients experienced AFL recurrence and 33% developed AF. The median time to AF occurrence was 7.4 months (interquartile range: 0.9–24.4 months). As compared with patients in the OAC discontinuation group, patients in the OAC continuation group had greater prevalence rates of prior AF and deep vein thrombosis. **Table 1** compares the baseline characteristics between the two groups.

During the study period, one patient (CHA_2_DS_2_-VASc score: 2 points) in the OAC discontinuation group without any history of AF experienced stroke 32 months after CA; at this point, there was also no AF detected, and the stroke was deemed atherosclerotic by imaging. No thromboembolic events in the OAC continuation group occurred. There were three major bleeding events in the OAC continuation group but none in the OAC discontinuation group (p < 0.001). All bleeding events were gastrointestinal in nature and required endoscopy and/or a blood transfusion. A comparison of outcomes of bleeding and thromboembolic complications between the two groups is shown in **Table 2**. In the OAC discontinuation group, 12 (20.3%) patients developed AF during follow-up and all but three of these patients were restarted on OAC. Reasons to refrain from starting OAC in these three patients were high bleeding risk, patient preference, and a short-duration AF episode following surgery. The mean time to occurrence of AF was 30.2 ± 26 months **(Figure 1B)**. The mean CHA_2_DS_2_-VASc score of these patients was 2.6 ± 1.7 points. Nine patients were restarted on OAC with a mean duration of 30.1 ± 24.6 months between OAC discontinuation and resumption.

## Discussion

In this single-center observational study, a strategy of OAC discontinuation with serial rhythm monitoring following successful AFL ablation appears to be safe and feasible. OAC discontinuation was associated with a significant reduction in major bleeding events with no increase in ischemic adverse events. Only one patient (1.7%) in the OAC discontinuation group experienced stroke and their imaging revealed an atherosclerotic origin, while cardiac monitoring failed to capture any recurrence of AFL or new-onset AF. OAC was stopped in 32.1% of patients at six months and in 55.7% at one year, likely as a result of heterogeneity between patients in terms of when the six months’ monitoring was done, secondary to delayed follow-up in a proportion of patients.

Current guidelines recommend continuing OAC indefinitely following AF ablation, according to established estimators of stroke risk such as the CHA_2_DS_2_-VASc score^1^; however, they do not take into account the perceived success of the ablation. In addition, there is significant practice variation, with prior studies reporting rates of OAC discontinuation as high as 62.5% at 12 months after ablation in patients with CHA_2_DS_2_-VASc scores of two points or more.^3^ In our study, patients in the OAC discontinuation group had a mean CHA_2_DS_2_-VASc score of 2.9 ± 1.4 points.

A major reason for continued OAC following AFL ablation is the occurrence of AF, with the incidence of AF ranging up to 50%.^4^ AF occurring after AFL ablation can increase the long-term stroke risk.^5^ However, a majority of these occurrences are usually in patients with a prior history of AF or LV dysfunction.^6^ This was apparent in our experience as well, where 44.3% of patients were on continued OAC with the predominant reason being a prior or new diagnosis of AF.

A risk of bleeding is one of the major reasons for possibly discontinuing OAC after AFL ablation. In our study, patients in the OAC discontinuation group had a significantly lower risk of developing major bleeding; this is consistent with findings of previous studies involving patients after AF ablation in which OAC discontinuation was associated with a lower risk of serious bleeding and a low incidence of ischemic strokes.^2,7^

### Study limitations

Our study is limited by its retrospective nature. The absence of continuous monitoring may also have led to the under-recognition of asymptomatic or intermittent episodes of AF. Using insertable cardiac monitors can potentially guide OAC decisions and present an attractive venue for targeted OAC. This has been previously studied in patients undergoing AF ablation, in which 68% of patients remained off OAC using an insertable cardiac monitor strategy.^8^ Another major limitation is the small sample size and the low event rate of stroke, which makes the study underpowered to draw conclusions regarding hard outcomes.

## Conclusion

In summary, we report successful OAC discontinuation in approximately half of our population that underwent successful AFL ablation. OAC discontinuation was associated with a significant reduction in major bleeding events. Periodic monitoring for incident AF following AFL ablation is crucial if an OAC discontinuation strategy is contemplated. Further studies examining rhythm-guided OAC can minimize unnecessary exposure to long-term anticoagulants.

## Figures and Tables

**Table 1:** Baseline Characteristics of the Study Population

	OAC Continuation (n = 47)	OAC Discontinuation (n = 59)	p-value
Age, mean ± SD	63.8 ± 11.8 years	64 ± 14.9 years	0.93
Male sex, n (%)	35 (74.5%)	48 (81.4%)	0.39
HTN, n (%)	32 (68%)	49 (83.1%)	0.07
DM, n (%)	13 (27.7%)	29 (49.2%)	< 0.05
CAD, n (%)	16 (34%)	14 (23.7%)	0.24
CHF, n (%)	14 (29.8%)	20 (33.9%)	0.65
COPD, n (%)	8 (17.0%)	5 (8.5%)	0.18
OSA, n (%)	9 (19.1%)	17 (28.8%)	0.25
Hyperthyroidism, n (%)	4 (8.5%)	1 (1.7%)	0.1
CKD, n (%)	6 (12.8%)	13 (22.0%)	0.23
History of AF, n (%)	20 (45.6%)	14 (23.7%)	< 0.05
History of VTE, n (%)	6 (12.8%)	1 (1.7%)	< 0.05
History of stroke, n (%)	6 (12.8%)	4 (6.8%)	0.3
CHAD_2_VASc score, mean ± SD	2.6 ± 1.3 points	2.9 ± 1.4 points	0.27
Tobacco use, n (%)	32 (68.1%)	31 (52.5%)	0.14
Alcohol use, n (%)	18 (38.3%)	19 (32.2%)	0.2
BMI, mean ± SD	29.9 ± 6.1 kg/m^2^	32.5 ± 7.3 kg/m^2^	0.06

AF: atrial fibrillation; BMI: body mass index; CAD: coronary artery disease; CHF: congestive heart failure; CKD: chronic kidney disease; COPD: chronic obstructive pulmonary disease; DM: diabetes mellitus; HTN: hypertension; OAC: oral anticoagulation; OSA: obstructive sleep apnea; SD: standard deviation, VTE: venous thromboembolism.

**Table 2:** Major Bleeding and Thromboembolic Events in the Study Population

	OAC Continuation (n = 47)	OAC Discontinuation (n = 59)	p-value
Hospitalization in 30 days, n (%)	10 (21.3%)	4 (6.8%)	< 0.05
Stroke, n (%)	0 (0%)	1 (1.7%)	0.38
Major bleeding event, n (%)	3 (6.4%)	0	< 0.05
AFL recurrence, n (%)	3 (6.4%)	0	< 0.05
AF after ablation, n (%)	23 (48.9%)	12 (20.3%)	< 0.05
Time to AF diagnosis	16.2 ± 42.2 months	30.2 ± 26 months	0.31

AF: atrial fibrillation; AFL: atrial flutter; OAC: oral anticoagulation.
